# Use of systemic antifungal drugs in critically ill patients. data from the envin-helics registry 2013-2014

**DOI:** 10.1186/2197-425X-3-S1-A398

**Published:** 2015-10-01

**Authors:** N Mas, P Olaechea, M Palomar, F Álvarez-Lermo, S Otero, S Uriona, M Catalán, ENVIN-HELICS Group

**Affiliations:** Intensive Care Unit, Hospital Galdakao-Usansolo, Galdakao, Spain; Intensive Care Unit, Hospital Arnau de Villanova, Lleida, Spain; Intensive Care Unit, Hospital del Mar, Barcelona, Spain; Department of Preventive Medicine, Hospital Vall d'Hebron, Barcelona, Spain; Intensive Care Unit, Hospital 12 de Octubre, Madrid, Spain

## Introduction

Flora in ICUs has changed with the development of new therapies and the implementation of protocols regarding bacterial infections. As a result, this could increase our concerns about fungal infections and modify the indications and the way we use them.

## Objectives

To assess the indications for the use of antifungal drugs (AD) during the 2013-2014 period using data from the ENVIN-HELICS registry.

## Methods

Data regarding the use of AD in patients admitted to ICU for over 24 hours is gathered. Different kinds of amphotericin are grouped (deoxycholate, liposomal and lipid) (AMP), as well as echinocandins (caspofungin, micafungin and anidulafungin) (ECH). Fluconazole (FLU) and voriconazole (VOR) are considered as different groups. Data from the 3 months of each year when the registry is complete is used. The indications are divided in prophylaxis, treatment of community-acquired infections, treatment of in-hospital acquired infection (out of ICU) and in-ICU acquired infection treatment. Whether the AD is started as a specific therapy or empirically is also studied; in the latter the microbiological results (positive or negative), the adequacy, modifications of AD, AD combination and sequential therapies are analyzed. Results are shown as percentages of the used AD.

## Results

2417 patients were treated with AD. 400 received more than one AD and 72 of them more than 2. Overall in-ICU mortality was 36.4%. Length of stay average was 18.7 ( ± 19.3 ) days. First choice of AD is shown in the table.

Of 1481 cases where the AD is started empirically, the adequacy is confirmed in 21.3%; in 45.0% of the cases the cultures were negative or were not collected; in 15.8% the infection is not confirmed. Mortality of those patients treated with more than one AD was 60.0%. In 62 cases (2.5%) both agents were initiated at the same time, but only in 25 of them the combination was used for more than 48 hours. The most frequently used combination was VOR plus caspofungin (32.0%). 260 patients (10.7%) received sequential therapy; these changes were justified as adjustment of the spectrum (33.2%), worsening of the clinical status (31.6%), toxicity (8.6%), development of resistance during the AD use (0.5%) and other reasons (12.3%). Among the 59 cases were the AD was switched due to a worsening of the clinical status, 51 were initially treated with FLU (86.4%) and switched to ECH in 45 cases (76.2%). Mortality in ICU in the latter group was 59.3%.

## Conclusions

AD use in critically ill patients is described. FLU was the most frequently used AD. Microbiological confirmation was scarce. Combined AD therapy was rarely used, while sequential AD were more frequently administered (10.7%). The switch to FLU was seen in order to reduce the spectrum of the therapy, whereas ECH were the choice when the clinical status worsened; mortality in the latter group was high.

**Table 1:** First choice of AD according to indication.

	**Total (n), (%)**	**Empirical (%)**	**Prophylaxis (%)**	**Community-acquired infection (%)**	**Out of ICU acquired infection (%)**	**In-ICU acquired infection (%)**
Fluconazole	1296, (53.6)	48.5	63.5	48	47	61
Echinocandins	807, (33.4)	39.2	25.9	32.8	37.7	31.7
Amphotericin	89, (3.7)	3.3	3.4	5.9	3.9	2
Voriconazole	225, (9.3)	8.9	7.1	13.2	11.3	5.1
Total (n), (%)	2417, (100.0)	1481,(61.3)	351,(14.5)	454,(18.8)	926,(38.3)	686,(28.4)

